# Impact of Aqueous Extract of *Arbutus unedo* Fruits on Limpets (*Patella* spp.) Pâté during Storage: Proximate Composition, Physicochemical Quality, Oxidative Stability, and Microbial Development

**DOI:** 10.3390/foods9060807

**Published:** 2020-06-19

**Authors:** Joaquina Pinheiro, Sidónio Rodrigues, Susana Mendes, Paulo Maranhão, Rui Ganhão

**Affiliations:** 1 MARE—Marine and Environmental Sciences Centre, ESTM, Polytechnic Institute of Leiria, 2520-641 Peniche, Portugal; susana.mendes@ipleiria.pt (S.M.); paulo.maranhao@ipleiria.pt (P.M.); rganhao@ipleiria.pt (R.G.); 2 MARE—Marine and Environmental Sciences Centre, Polytechnic Institute of Leiria, 2520-630 Peniche, Portugal; sidoniojcrodrigues@gmail.com

**Keywords:** strawberry tree, molluscs, antioxidant, phenolic, colour, shelf-life

## Abstract

Limpets are molluscs widely used in food diet and much appreciated in many regions. The consumption of fishery products rich in polyunsaturated fatty acids has been increasing through filleted products and restructured products. Since food oxidation is the major cause of nutritional quality deterioration in fish products, the interest in the replacement of synthetic antioxidants with natural sources, namely in the preparation of restructured animal products such as burgers, sausages and pâtés, has been increasing. Phenolic compounds from fruits and vegetables have recognised antioxidant properties and are therefore currently considered as good alternatives to synthetic antioxidants in the food industry. In this study, the effects of the extracts of *Arbutus unedo* fruits, at two concentration levels (3% and 6%), on proximate composition, physicochemical properties, oxidative stability and safety of limpets pâté, during 90 days at refrigerated storage, were investigated. After processing, the addition of 3% and 6% of *A. unedo* extracts into limpets pâté contributed to an increase of 18% and 36% in the total phenolic content and 5% and 36% in the antioxidant capacity, respectively. During storage, the enriched limpets pâté with *A. unedo* fruit extracts at 6% was more efficient as an enhancer of oxidative stability, with 34% inhibition of lipid oxidation, highlighting the potential use of *A. unedo* fruits as a functional ingredient in the fish industry. Overall, the limpets pâté with 6% of *A. unedo* fruit extracts proved to be more efficient regarding microbial control, and had the lowest changes in the quality parameters such as in colour, texture and pH during 90 days at refrigerated storage.

## 1. Introduction

In Portugal, a traditional country of seafood consumption due to its geographical location and ocean access allied with the promotion of health benefits, a high value was achieved, around 56.8 kg/capita/year compared with the European average (24.9 kg/capita/year) [1]. This healthy diet is related mainly to the nutritional abundance such as vitamins, digestible protein, n-3 long chain polyunsaturated fatty acids (n-3 LC-PUFAs) and minerals (iodine, selenium) [2]. However, the higher demand of these products has led to reductions in natural stocks, especially in fish species like cod, sardine and hake [3]. On the other hand, several underutilised fish due to an inappropriate colour or texture and some fish by-products can be transformed into high-value products [3]. In this context, other less exploited marine resources need to be used to facilitate the guarantee of sustainable development and integrity of the marine environment. New seafood products, such as transformed/restructured products, provide an excellent opportunity to improve endogenous products and contribute to the high added value of food, allowing its consumption.

Limpets (*Patella* spp.) are gastropod molluscs and common species in the rocky Mediterranean area and their consumption has been appreciated in several regions of Portugal. The limpet’s usage constitutes an added value for a food product such as pâté, which is usually made with a high content of animal fat [4]. Since they have a high content of polyunsaturated fatty acids rich in omega-3, omega-6 and omega-9 that cannot be synthesised by the human body, limpets can be included in the diet for the prevention of cardiovascular, cancer and inflammatory diseases. Pâté is a minced muscle-based food, traditionally elaborated with meat (goose or pork liver) and mixed with water and different additives, which is manufactured, packaged in a glass container and heat-treated [5]. The main problem of this food product is lipid oxidation due to the presence of a higher fat content and low natural antioxidants [6], resulting in polyunsaturated fatty acids degradation with lipid-derived volatiles emergence and the generation of residual products, such as malondialdehyde (MDA), leading to the deterioration of nutritional and sensory quality. Particular animal food products consumed worldwide, such as burgers, patties and sausages, are considerably susceptible to oxidation by mincing and cooking, promoting the formation of reactive oxygen species (ROS) and hence the occurrence and intensity of oxidative reactions [7,8].

The incorporation of synthetic antioxidant additives such as 2,6-di-tert-butyl-4-hydroxytoluene (BHT) is widely used in the food industry to prevent this food problem [6]. However, there is a high demand for food without synthetic ingredients, leading to the study of healthy food compounds, such as natural antioxidants from plants. Nowadays, natural antioxidants are employed in food products because of their potential health benefits and safety. Therefore, a growing interest in the identification of novel and natural antioxidants for developing functional foods with enhanced nutritional and health properties is emerging. The antioxidant activity of fruit extracts is important thanks to their beneficial physiological activity on human cells, and their potential to replace synthetic antioxidants, used in foodstuffs [9,10]. The antioxidant potential of berries from Scandinavian forests against muscle lipid oxidation has been profusely documented [11] compared with the potential use of certain Mediterranean berries that has been less investigated. Recent studies have proved the antioxidant potential, by preliminary tests, on the oxidative stability of meat products of one endemic species of Portugal, the strawberry tree’s (*Arbutus unedo*, Ericaceae family) fruits [12,13,14,15]. However, there is still a lack of information regarding their effects on the oxidative stability of fish and fish products. Moreover, the *A. unedo* fruits represent an important resource for populations as an alternative to common resinous species to reforestation and in food preparation such as in the traditional Portuguese liquor “Aguardente de Medronho”, marmalades, jams, jellies or yogurts [12]. Furthermore, over the years, the health benefits of *A. unedo* fruits have been applied in traditional medicine, revealing antidiabetic and anti-tumoral properties, and are also used to treat cardiovascular diseases such as thrombosis, atherosclerosis and hypertension [16,17]. These properties are related to the natural phytochemicals present in berries such as phenolic compounds, vitamins C and E and carotenoids [18,19]. Based on the scientific literature, the *A. unedo* fruits show an interesting proximate composition, where the highest macronutrient compounds, with the exception of moisture (59.70 g/100 g) [18], were the carbohydrates fraction (23.55 g/100 g) and dietary fibre (16.21 g/100 g) [19], followed by protein (30.9 ± 0.8–33.6 ± 1.2 g/kg of dry weight) and fatty acids, where α-linolenic acid, oleic acid and linoleic acid themselves had a prominent role [18].

The aim of the present study was to evaluate the proximate composition of limpets pâté with the natural antioxidant extract from *A. unedo* fruits at two concentration levels (3% and 6%) compared with a control limpets pâté (BHT). All the pâté samples were assessed by a physicochemical, oxidative stability and microbial evaluation during 90 days at refrigerated storage.

## 2. Materials and Methods

### 2.1. Location and Limpets Handling

Limpets were collected from the western coast of Portugal at the beach of *Portinho da Areia Norte* (Peniche). The removal of limpets from the rocks was performed by knife and then they were transported in sea water to the laboratory for preparation: washing with salt water, shell removal, sand fragments with the dowel, re-washing with salt water, draining the core for approximately 30 s, weighting, sealing in a vacuum bag and storing at −80 °C until processing.

### 2.2. Location and Preparation of Arbutus unedo Fruits Extracts

The *A. unedo* fruits were harvested in Autumn in the center region of Portugal at the full ripeness stage and transported to the laboratory for preparation. The fruits were selected, cleaned, sorted to eliminate damaged and shrivelled fruits, weighted, sealed in a vacuum bag and frozen at −80 °C until the antioxidant extract preparation. The antioxidant extraction was performed in accordance with the modified method reported by Ganhão et al. [14]. The fruits were cut into small pieces, 30 g of fruits were weighed, transferred to a Falcon tube and freeze-dried at −60 °C until a constant weight (up to 72 h). Afterwards, the freeze-dried fruits were homogenised with 300 mL of water (1:10, *w*:*v*) using a homogeniser (Velp Scientifica, Usmate, Italy). The homogenates were centrifuged at 4000 rpm for 10 min at 6 °C using the centrifuge 5810 R (Eppendorf, New York, NY, USA). The supernatants were filtrated, collected and the residue was re-extracted once more following the procedure previously described. The two supernatants were combined and stored under refrigerated conditions until analysis (<24 h).

### 2.3. Evaluation of Proximate Composition, Physicochemical Properties, Oxidative Stability and Microbial Development of Limpets Pâté with Natural and Synthetic Antioxidant Additives

#### 2.3.1. Processing of Limpets Pâté Enriched with Natural and Synthetic Antioxidant Additives

The limpets pâté was formulated according to the processing methods described by Estévez et al. [20], Sánchez-Zapata [5] and Nielsen and Jacobsen [21], with some modifications.

The experimental limpets pâtés were prepared in a pilot plant. The standard pâté was formulated using 62% limpets, 12% water, 10% milk, 8% oil, 7% potato starch, 1% margarine, 0.003% salt (sodium chloride), 0.002% white pepper and 0.002% nutmeg. The limpets pâté samples were prepared according the addition of antioxidant compounds: BHT at 0.01% (ID: CTR), with the *Arbutus unedo* fruits extract at 3% (ID: PAU3) and 6% (ID: PAU6) (see Table S1 in Supplementary Material).

Depending on the experimental batch, different antioxidant compounds were added to the standard formula. The natural antioxidants are generally recognised as safe and were added at two levels: 3% and 6% [14]. The synthetic antioxidant butylated hydroxytoluene (BHT) was added according to the Portuguese law and the food product (0.01%) [22].

The limpets pâté batches were manufactured as follows: firstly, the limpets’ cores were steam heat-treated at 100 °C for 10 min in an electric oven (Foinox, MM 100 E Ecomix, Codogné, Italy), and chopped in a cutter (Robot coupe, R8 V.V, Montceau-en-Bourgogne-Cedex, France) until obtaining a homogeneous limpets paste, firstly at 1500 rpm for 3 min and then at 2000 rpm for 3 min. After this process, the potato starch mixture and oil were added until obtaining a homogeneous paste (3000 rpm for 5 min) and the pâtés were packaged in glass containers and subjected to heat treatment at 80 °C for 30 min in a water bath and cooling at room temperature before being stored at refrigerated temperature (5 °C) for 90 days in the dark. The limpets pâtés were analysed at days 0, 30, 60 and 90 regarding the physicochemical, phytochemical, microbial and oxidative stability. At the sampling times, instrumental colour and texture were measured on the surface of the limpets pâtés and then the samples were stored at 5 °C until the other analytical experiments were conducted.

#### 2.3.2. Proximate Composition

For the determination of the proximate composition (moisture, protein, fat, carbohydrate, ash and fibre) of the limpets pâté samples, the AOAC methods [23] were followed.

The moisture content of the pâté samples was determined by drying the homogenised sample in an oven (Binder, Bohemia, NY, USA) at 103 °C until a constant weight. The moisture was calculated as follows:

(1) $$\mathrm{Moisture}\;(\%)=\left(\frac{\mathrm{wet}\;\mathrm{weight}\;-\;\mathrm{dry}\;\mathrm{weight}}{\mathrm{wet}\;\mathrm{weight}}\right)\;\times \;\;100$$

The crude protein was determined by the Kjeldhal method (N × 6.25). Briefly, 1 g of sample was digested in Kjeldhal digestion flasks with 20 mL of concentrated sulphuric acid and 15 g of Kjeldhal catalyst. The digestion flask was heated at 380 °C for 45 min in a Kjeldahl digester (FOSS, Hillerod, Denmark). The flask was allowed to cool at room temperature and the distillation was carried out in a Büchi Distillation Unit (mod. B-324) (Buchi, New Castle, DE, USA) and 0.1 N HCl standard solution was used as the titration acid. Fat was determined by extracting 5 g of sample with petroleum ether using a Soxhlet apparatus (Behr, Labor Technik, Düsseldorf, Germany), ash content was obtained by incineration at 600 ± 15 °C and carbohydrates were calculated by the difference as follows:

(2) Carbohydrates (%)=100 − (g moisture+g protein+g fat+g ash)

Moreover, the total energy was calculated using the following equation:

(3) Energy (kcal)=4 × (g protein+g carbohydrate)+9 × (g fat)

The crude fibre was determined by acid and basic digestion of 1 g of defatted sample that was added to 150 mL of sulfuric acid (1.25%), then it was stirred, boiled for 30 min and filtered with Whatman no. 4. Afterwards, the basic digestion was realised, in the same proportions of the solvents using sodium hydroxide (1.25%) and the remained residue. After, the residue was dried at 105 °C for 8 h until the weight was constant, following drying at 105 °C (until constant weight), and ashed in a muffle at 550 °C for 5 h, cooled and weighted. The difference between the ash weight subtracted from the weight of the insoluble matter was expressed as the crude fibre percent of the original weight content.

#### 2.3.3. Physicochemical Quality

The limpets pâté samples were analysed for colour, texture and pH value. All analyses were performed in triplicate on days 0, 30, 60 and 90 of storage.

Colour of all pâté samples was evaluated by a tristimulus colourimeter (Minolta chroma Meter, CR-400, Osaka, Japan). The instrument was calibrated using a white standard tile (L* = 97.10, a* = 0.19, b* = 1.95), illuminate D65 and observer 2°. Commission Internationale de l’éclairage (CIE) colour space coordinates, the L*a*b* values, were determined in all pâté samples in a Petri dish, previously filled, and three measurements per sample were performed at room temperature (≈20 °C). L* values represent the luminosity of the samples (0—black to 100—white), and a* and b* values indicate the variation of greenness to redness (−60 to +60) and blueness to yellowness (−60 to +60), respectively.

Texture was determined according to the modified method described by Estévez et al. [24]. The penetration test was performed using a Texture Analyser (TA.HDi, Stable Microsystem Ltd., Godalming, UK) using a 30 kg load cell and a stainless steel cylinder probe with a 10 mm diameter. The penetration test was realised at 1.5 mm.s^−1^ of speed and 8 mm of penetration distance. Firmness (maximum peak force (N)) and adhesiveness (N/s) were used as indicators of the texture parameter. Firmness was measured at room temperature (~22 °C) to avoid storage temperature effects on analysis.

The pH of all pâté samples was measured at room temperature using a pH meter (SP70P, SympHony, Radnor, PA, USA).

#### 2.3.4. Antioxidant Capacity and Oxidative Stability

Total phenolic content (TPC) was determined by the Folin–Ciocalteu method reported by Yu et al. [25], with slight modifications. An amount 10 μL of sample/standard was mixed with 790 μL water and 50 μL of Folin–Ciocalteu reagent. After 2 min, 150 μL Na_2_CO_3_ solution (20%, *w*/*v*) was added. After the occurrence of a reaction at room temperature in the dark (60 min), the absorbance of the mixture was measured at 755 nm (BioTek Instruments, Winooski, VT, USA). Gallic acid was used for the standard calibration curve. The analysis was performed in triplicate and the results were expressed as mg of gallic acid equivalents per grams of sample (mg GAE/g). The analyses were made on storage days 0, 30, 60 and 90.

DPPH radical scavenging activity was determined by the decrease in DPPH radical absorption after exposure to radical scavengers [26], according to the modified method described by Duffy and Power [27]. An amount of 10 μL of sample/standard was mixed with 990 μL of DPPH solution (0.1 mM). The reaction mixture was vortexed and allowed to stand during 30 min in the dark at room temperature. After, the absorbance was measured at 517 nm (BioTek Instruments, Winooski, VT, USA), the radical scavenging activity was calculated as a percentage of the DPPH discolouration using the following equation:

(4) $$\mathrm{DPPH}\;\mathrm{radical}\;\mathrm{scavenging}\;\mathrm{activity}\;(\%)=\left(\frac{A_{0}\;-\;A_{1}}{A_{0}}\right)\;\times \;100$$

where *A*_0_ is the absorbance of the DPPH solution and *A*_1_ the absorbance of the DPPH radical and sample.

The DPPH radical scavenging activities of all pâté samples were determined on storage days 0, 30, 60 and 90.

Lipid oxidation of the limpets pâté was evaluated according to the thiobarbituric acid reacting substances (TBARs) assay reported by Rosmini et al. [28], with some modifications. An amount of 15 g of each pâté sample was homogenised with the solution of trichloroacetic acid at 7.5%, propyl gallate and ethylenediamine tetra acetic acid (EDTA) and then was filtrated by Whatman no. 4 filter paper. Next, 5 mL of TBA was added to the filtrate, vortexed and incubated in a boiling water bath at 100 °C for 40 min. After cooling, the absorbance was measured at 530 nm (BioTek Instruments, Winooski, VT, USA). The standard curve was prepared using a 1,1,3,3-tetraethoxypropane (TEP) solution and results were expressed as mg malondialdehyde (MDA) per kg of limpets pâté. The analyses were made on storage days 0, 30, 60 and 90.

Further, the percent of inhibition against lipid oxidation was calculated at day 90 as follows:

(5) $$\mathrm{Inhibition}\;\mathrm{lipid}\;\mathrm{oxidation}\;(\%)=\left(\frac{C_{90}\;-\;T_{90}}{C_{90}}\right)\;\times \;\;100$$

where *T*_90_ is the amount of MDA in the treated pâté at day 90 and *C*_90_ is the amount of MDA in the control sample of pâté at day 90.

#### 2.3.5. Microbial Development

Microbial development was performed according to European standard legislation [29].

Twenty-five grams of samples and 225 mL of peptone water were transferred into sterile stomacher filter bags and homogenised in a stomacher (Interscience, St Nom, France) at speed 8 for 10 min. After, a ten-fold dilution series was prepared and plates containing the agars were inoculated. Total viable mesophilic counts [30] were determined using plate count agar (PCA) and incubated at 30 ± 1 °C for 72 h. *Escherichia coli* [31] was carried out using TBX agar and incubated at 37 °C ± 1 °C for 4 h and 44 °C between 18 and 20 h. *Listeria monocytogenes* [32] was determined using *Rapid L. mono* and incubated at 35–37 °C for 48 h. *Salmonella* spp. [33] was performed using *Samonella Express* and incubated at 41.5 ± 1 °C for 24–26 h.

The results were expressed as the logarithm of colony-forming units per gram of limpets pâté sample (Log_10_ cfu/g). The analyses were made on days 0, 30, 60 and 90 of storage.

### 2.4. Statistical Analysis

For each limpets pâté, three samples were performed, and all the analysis were carried out in triplicate. All data were checked for normality and homoscedasticity. A one-way analysis of variance (ANOVA) with Dunnett’s multiple comparison of group means was employed to determine significant differences relative to the control pâté. In the remaining conditions, multiple comparisons were performed using Tukey’s honestly significant difference (HSD). When the homogeneity of the variance and normality of the data were not fulfilled, the nonparametric Kruskal–Wallis test [34] was used, followed by the Games–Howell non-parametric post-hoc test. In addition, to evaluate the strength of the correlations between the quality attributes, Pearson’s correlation coefficient (r) was used. The calculations were performed using the Statistica™ v.8 Software from Stasoft [35]. All results were considered significant at the *p*-value < 0.05 level. The results are presented as mean ± standard deviation (SD).

## 3. Results and Discussion

### 3.1. Evaluation of Proximate Composition of Limpets Pâté

The proximate composition (moisture, protein, fat, carbohydrate, fibre, ash) of the limpets pâté samples with synthetic (BHT) and natural (*Arbutus unedo* fruits extracts) antioxidant sources is shown in Table 1.

The addition of *A. unedo* fruits extracts at 3% and 6% did not significantly (*p*-value > 0.05) influence the nutritional composition of the standard limpets pâtés, since similar values of moisture, fat, protein, carbohydrate, ash and energy values, between samples, were found. This similar nutritional profile may result from the same experimental formula used for all pâté samples with the exception of the inclusion of different concentrations of antioxidants ingredients. Aquerreta et al. [36] found an identical proximate composition in fish pâtés elaborated with different quantities of tuna liver and mackerel flesh and also in commercial fish pâtés elaborated with tuna, large-scaled scorpion fish, salmon and anchovy, varying between the range of 164 and 301 kcal/100 g. As stated by previous studies, the traditional pâté usually prepared with goose/pork denotes a higher calorific value, near to 450 kcal/100 g [37], when compared with fish pâté.

The crude fibre content significantly (*p*-value < 0.05) increases after the inclusion of 3% and 6% of *A. unedo* fruits extracts in the limpets pâté formulation to 0.2% and 0.80%, respectively. This increment can be explained by the highest source of crude fibre revealed by the *A. unedo* fruits, 15.4 ± 0.8% (see Table S2 in Supplementary Material) and previously reported by Barros et al. [26] and Morgado et al. [18].

### 3.2. Evaluation of Physicochemical Quality of Limpets Pâté

The effects of *Arbutus unedo* fruits extracts on the colour, texture and pH value of the limpets pâté during 90 days of storage at 5 °C are shown in Table 2.

Considering the colour of the limpets pâté samples, the incorporation of *A. unedo* fruits extracts induced a luminosity decrease in both enriched limpets samples compared with CTR. However, only the highest concentration of fruits extract promoted a significant (*p*-value < 0.05; Table 2) browning of the limpets pâté compared with the luminosity observed in the CTR sample. In the study reported by Estévez et al. [38], no significant differences (*p*-value > 0.05) in luminosity between batches of porcine liver pâtés elaborated with sage and rosemary essential oils, as antioxidants, were detected.

During storage, an increase in luminosity in all pâté samples was evident wherein the PAU3 and PAU6 samples revealed the smallest and highest values, 48.3 ± 0.7 and 55.2 ± 0.7, respectively, at the end of storage. As previously mentioned by Sánchez-Zapata et al. [5], the luminosity is a colour parameter highly influenced by water, fat, collagen content and free water on the product surface, so an increase in luminosity was expected during the storage period.

Regarding the colour coordinates of a* and b* (Table 2), no significant statistical differences (*p*-value > 0.05) were observed between the limpets pâté samples, with the values ranging from 1.2 to 1.8 and 27.1 to 33.0, respectively. According to Agregán et al. [6], the richness of the antioxidants of *A. unedo* fruits leads to a good stabilisation of the food’s colour, as observed in all limpets pâté samples, with natural antioxidant extracts from *A. unedo* (PAU3, PAU6) and synthetic (BHT) ones.

Another physical parameter with interest from the consumer point of view is the texture. Pâté is considered a paste-like texture composed of a mixture of proteins, fat, water, salt and spices that when mixed result into a homogeneous mass [39]. The *A. unedo* extracts produced a statistically significant effect (*p*-value < 0.05; Table 2) in the texture of the limpets pâté samples. Comparing the maximum force of the CTR samples, both enriched pâté samples exhibited a significant (*p*-value < 0.05; Table 2) decrease of 12% and 35%, in PAU3 and PAU6, respectively. The storage time influenced the hardness of all pâté samples, where an augment of 13% was observed on PAU3, followed by PAU6 (14%) and CTR (23%) after 90 days of storage. The increase in hardness in the pâté samples comes out from separation of the water and fat of the protein matrix due to emulsion destabilisation as stated by Fernández-López et al. [40] and Amaral et al. [41] in ostrich liver pâté. Moreover, the richness of *A. unedo* fruits in fibre (see Table S2 in Supplementary Material) can contribute to an increase in firmness of limpets pâtés.

The pH value of limpets pâté samples during 90 days of storage are shown in Table 2. Despite the identical pH value of all pâté samples after processing, a slight and significant decrease (*p*-value < 0.05, Table 2) was observed with the incorporation of *A. unedo* fruits extracts at both concentration levels, 3% and 6%, compared with the CTR sample. The decrease in pH in all enriched limpets pâtés can be due to the richness of *A. unedo* fruits in organic acids, as for example, the presence of fumaric (1.49 mg/g), lactic (0.49 mg/g), malic (0.84 mg/g) and citric (0.01 mg/g) acids [42]. This fact can promote the retention of antioxidants [43] in a natural way, through the extract of *A. unedo*. Moreover, during storage, a similar (*p*-value > 0.05, Table 2) pH value was detected in all pâté samples, being the lowest and most significant value (*p*-value < 0.05, Table 2) encountered in both enriched limpets pâtés samples, contrary to the observed in the CTR sample. This increase in the pH value has been reported as a common behaviour in other stored pâtés [44,45,46]. The data suggest that *A. unedo* fruits extracts can be responsible for the initial pH changes on limpets pâté, contributing to the pH stability of the processed food product.

### 3.3. Evaluation of Antioxidant Capacity and Oxidative Stability of Limpets Pâté

**Figure 1:**
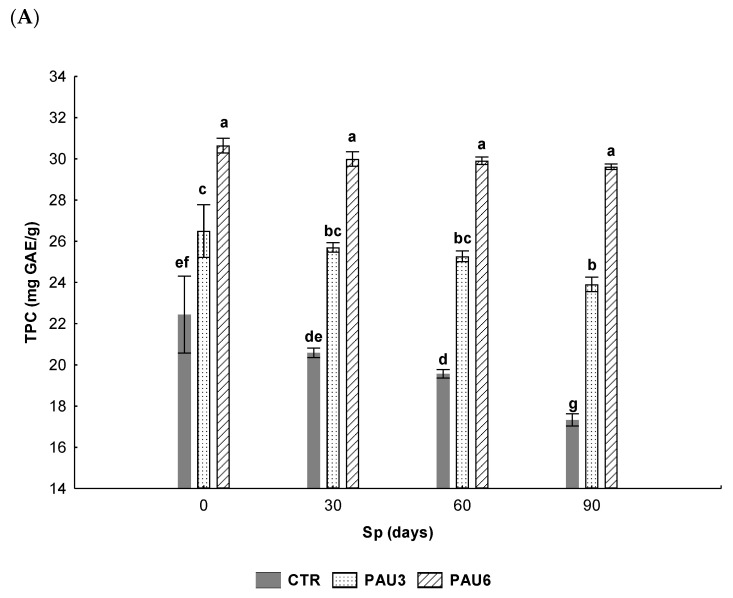

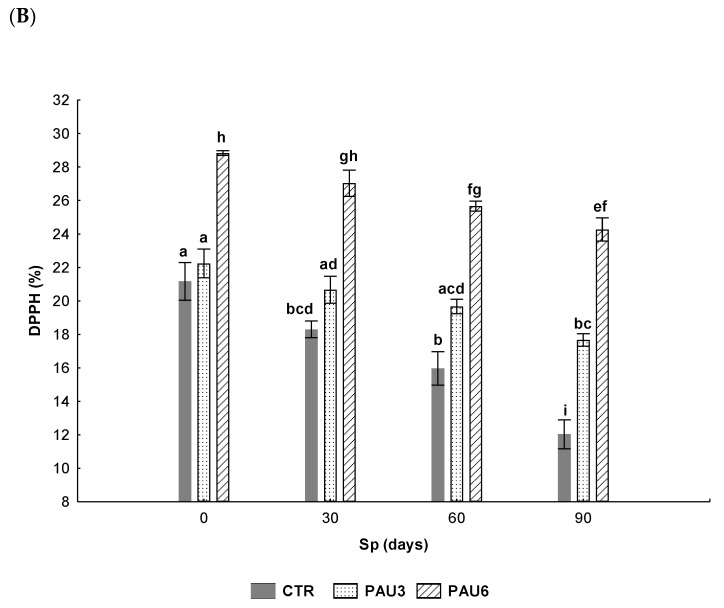
Effect of *A. unedo* fruits extracts in the total phenolics content (TPC) (**A**) and DPPH radical scavenging activity (**B**) of limpets pâté samples (CTR—with synthetic additive; PAU3—with 3% of *A. unedo* fruits extract; PAU6—with 6% of *A. unedo* fruits extract) stored at refrigerated temperature for 90 days. Bars with different letters represent significant differences (*p*-value < 0.05). Error lines represent the standard deviation.

The stability of limpets pâté samples enriched with *Arbutus unedo* fruits extract, as a natural antioxidant additive, at two concentration levels, was determined by the total phenolics content and DPPH radical scavenging activity. Through both methodologies, it was possible to observe an increase in phenolics content and DPPH radical scavenging activity in the PAU3 and PAU6 samples, compared with the CTR samples (Figure 1A,B).

The PAU6 limpets pâté sample showed the highest phenolic content (30.6 ± 0.4 mg GAE/g) followed by the PAU3 sample (26.5 ± 1.3 mg GAE/g) and CTR samples (22.4 ± 1.9 mg GAE/g) (Figure 1A). This effect reveals the richness in phenolic compounds (567± 27 mg GAE/g) of *A. unedo* fruits and is in agreement with other authors [19,26,27]. Further, the *A. unedo* fruits have been identified as a good source of antioxidants and can play an important role in human nutrition [19,47], being beneficial to health as antibacterial, anti-inflammatory and anti-carcinogenic agents. [26]. During storage, a similar behaviour was constated in all pâté samples, reaching the end of storage with the same trend value (PAU6 > PAU3 > CTR).

The results of antioxidant capacity expressed by DPPH radical scavenging of the limpets pâtés are presented in Figure 1B and a higher inhibition percentage was revealed in both samples enriched with *A. unedo* fruits extracts. At day 0, no significant differences (*p*-value > 0.05) were detected in the antioxidant capacity of the PAU3 sample compared with the CTR sample. However, this difference became increasingly significant (*p*-value < 0.05; Figure 1B) during refrigerated storage. As observed in the TPC evaluation, the inhibition of DPPH decreases about 43%, 20% and 15% in the CTR, PAU3 and PAU6 samples, respectively, at the end of storage. Arshad et al. [48] reported a similar behaviour, between TPC and DPPH scavenging activity, where a higher TPC led to a higher free radical scavenging activity.

A positive and linear correlation (r = 0.90; *p* < 0.05) between the two methodologies used for the antioxidant activity evaluation for all limpets pâté samples was found. This correlation is in line with those reported by Szydłowska-Czerniak et al. [49], which proved the benefit of the Folin–Ciocalteu reducing capacity method for the assessment of the total antioxidant capacity of food samples.

Lipid oxidation is one of the major problems concerning food quality deterioration, mainly in fish products due to the higher proportions of long chain unsaturated fatty acids making them more susceptible to oxidation [37]. Thiobarbituric acid reacting substances (TBARS) values represent the content of secondary lipid oxidation products, mainly aldehydes (or carbonyls) which contribute to the deterioration of quality attributes such as flavour, colour and texture in oxidised food products [49,50,51].

**Figure 2:**
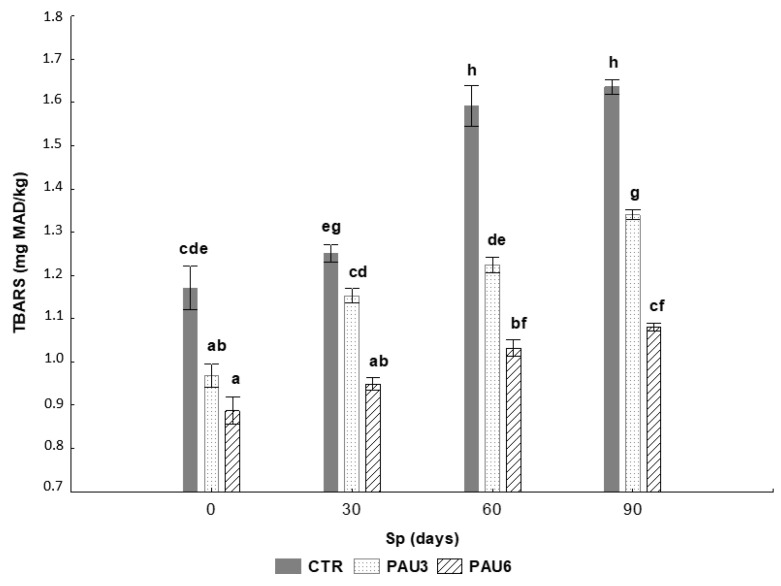
Effect of *A. unedo* fruits extracts on thiobarbituric acid reacting substances (TBARS) of limpets pâté samples (CTR—with synthetic additive; PAU3—with 3% of *A. unedo* fruits extract; PAU6—with 6% of *A. unedo* fruits extract) stored at refrigerated temperature for 90 days. Bars with different letters represent significant differences (*p*-value < 0.05). Error lines represent the standard deviation.

The effect of extracts from *A. unedo* fruits as natural inhibitors of lipid oxidation in the limpets pâté samples during 90 days at refrigerated storage is shown in Figure 2.

After processing, a significant decrease (*p*-value < 0.05; Figure 2) in the TBARS value in PAU3 and PAU6 (0.97 and 0.89 mg MAD/kg) was achieved when compared with the limpet pâté with synthetic BHT (1.17 mg MAD/kg). During storage, the TBARS values increased gradually on all the experimental analysis days, and the CTR and PAU6 limpets samples showed, always, the highest and lowest value, respectively. Ganhão, et al. [51] explained that the increase in malondialdehydes during refrigerated storage is a result of the onset of oxidative reactions and for this reason, this behaviour was expected on the limpets pâté. Further, the inhibition of lipid oxidation in PAU6 reached almost double the percentage obtained in PAU3, 34% and 18%, respectively. These results indicate a higher protection of limpets pâté against lipid oxidation with the addition of 6% of *A. unedo* fruits extract. The ability of this extract to inhibit the oxidative deterioration of fish products can be attributed to the antioxidant activity of the phenolic compounds naturally present in *A. unedo* fruits, which is in accordance with the results previously reported. It is plausible to consider that the phenolic compounds from this fruit inhibited the formation of TBARS through the protection of polyunsaturated fatty acids against reactive oxygen species (ROS).

Beyond the influence of lipid oxidation products on the sensory quality of fish products, MDA and other TBARS have been highlighted as mutagenic compounds with carcinogenic potential [52]. By inhibiting the formation of TBARS in pâtés, added fruit extracts might improve the overall quality of these products and increase the nutritional value from a health perspective.

### 3.4. Evaluation of Microbial Quality of Limpets Pâté

The effects of *Arbutus unedo* fruits extracts on the microbial development of the limpets pâté samples (CTR, PAU3, PAU6) along 90 days at refrigerated storage are shown in Table 3.

Immediately after processing, no microbial count was found in all pâté samples. The absence of microorganisms at this stage is expected and can be explained by the heat treatment applied during the limpets pâté processing. Only after 60 days at refrigerated storage, the viable mesophilic count was developed in all pâté samples. The addition of *A. unedo* fruits extracts on the limpets pâté samples at 3% and 6% conducted a reduced microbial development (4.3 and 4.2 Log_10_ cfu/g, respectively) compared with the CTR sample (4.8 Log_10_ cfu/g). Moreover, the microbial pathogenic species (*Escherichia coli*, *Salmonella* spp. and *Listeria monocytogenes*) were not detected along 90 days of storage on the pâté samples. According to Puupponen-Pimiä et al. [53], wild fruits such as *A. unedo*’s, are rich in phenolic polymers, like ellagitannins, that act as antibacterial agents. There are several mechanisms of action that promote the inhibition of microorganisms, such as the destabilisation of the cytoplasmic membrane, permeabilisation of the plasma membrane, inhibition of extracellular microbial enzymes, direct actions on microbial metabolism and deprivation of the substrates required for microbial growth [53]. The antibacterial effect of aqueous extracts from *A. unedo* fruits is in accordance with Malheiro et al. [16], who reported the antibacterial effect against *Escherichia coli*. Further, Salem et al. [54] reported the in vitro antimicrobial activity of ethanolic *A. unedo* fruits extracts against Gram-positive and Gram-negative bacteria as *Salmonella typhimurium*, *Escherichia coli* and *Enterococcus feacium*.

## 4. Conclusions

Based on the present study, one endemic species of Portugal, the strawberry tree, *Arbutus unedo*, and its fruits, considered as a potential natural additive, revealed a successful strategy to attain the prevention of lipid oxidation of a new food product, limpets pâté stored at refrigerated storage for 90 days. Regarding the microbial development and the requirements of food legislation, a safe food product was achieved at the end of storage. The nutritional composition of the limpets pâté did not change by the addition of *A. unedo* fruits extracts, as a natural additive and new ingredient, with the exception of the fibre content, where an increase was observed. According to the obtained results, the enriched limpets pâté with 6% of *A. unedo* fruits extracts emerged superior by the quality improvement and maintenance of the stored limpets pâté, mainly in the total phenolics content, antioxidant capacity, colour and texture of the stored limpets pâté. In summary, the obtained results demonstrated the benefits of the addition of a natural antioxidant ingredient, *A. unedo* fruits, by the achievement of oxidative stability and maintenance of quality in a new, natural and healthy food product, limpets pâté.

## Figures and Tables

**Table 1:** Proximate composition (mean ± SD (*n* = 3)) of limpets pâté samples without (CTR—limpets pâté with butylated hydroxytoluene (BHT)) and with *Arbutus unedo* fruits extracts (PAU3—limpets pâté enriched with 3% of *A. unedo* fruits extract, and PAU6—limpets pâté enriched with 6% of *A. unedo* fruits extract).

Proximate Composition	CTR	PAU3	PAU6
Moisture (%)	65.5 ± 0.2	65.5 ± 0.2	65.2 ± 0.1
Fat (%)	12.6 ± 0.2	12.7 ± 0.5	12.9 ± 0.4
Protein (%)	11.9 ± 0.0	11.8 ± 0.0	11.9 ± 0.1
Carbohydrate (%)	7.8 ± 0.4	8.1 ± 0.5	8.0 ± 0.6
Ash	2.2 ± 0.2	1.9 ± 0.2	2.0 ± 0.2
Fibre	0.0 ± 0.0 ^a^	0.2 ± 0.0 ^b^	0.8 ± 0.0 ^c^
Energy value (kcal/100 g)	192.2 ± 1.3	193.7 ± 2.5	195.5 ± 1.0

Different letters, in the same line, indicate significant differences at *p*-value < 0.05.

**Table 2:** Effects of *A. unedo* fruits extracts on colour (L*, a*, b*), texture (maximum force and adhesiveness) and pH of limpets pâté samples (CTR, PAU3 and PAU6; limpets pâté with BHT, with 3% and 6% of *A. unedo* fruits extract, respectively). The results are presented as mean ± SD (*n* = 3).

Limpets Pâté	Storage Period (days)	Colour			Texture		
		L*	a*	b*	Maximum Force (N)	Adhesiveness (N/s)	pH
**CTR**	0	34.5 ± 1.3 ^c^	1.3 ± 0.0 ^ac^	28.4 ± 0.9 ^ab^	4.3 ± 0.0 ^b^	−2.1 ± 0.1 ^c^	6.8 ± 0.0 ^c^
	30	42.7 ± 0.5 ^a^	1.4 ± 0.0 ^ab^	27.1 ± 0.5 ^a^	4.5 ± 0.1 ^b^	−2.1 ± 0.1 ^c^	6.9 ± 0.0 ^d^
	60	42.6 ± 0.8 ^a^	1.5 ± 0.1 ^ab^	28.8 ± 0.6 ^ab^	4.7 ± 0.1 ^f^	−2.5 ± 0.1 ^f^	6.9 ± 0.0 ^cd^
	90	47.0 ± 0.5 ^d^	1.6 ± 0.0 ^bde^	31.6 ± 0.5 ^cd^	5.3 ± 0.1 ^g^	−3.1 ± 0.1 ^e^	6.8 ± 0.0 ^c^
**PAU3**	0	33.2 ± 0.8 ^bc^	1.4 ± 0.1 ^ac^	27.7 ± 0.9 ^ab^	3.8 ± 0.1 ^d^	−1.4 ± 0.1 ^ad^	6.6 ± 0.0 ^b^
	30	43.2 ± 0.5 ^a^	1.7 ± 0.0 ^de^	27.7 ± 0.2 ^ab^	3.8 ± 0.1 ^de^	−1.4 ± 0.1 ^a^	6.6 ± 0.0 ^ab^
	60	44.4 ± 0.6 ^a^	1.7 ± 0.0 ^ef^	28.7 ± 0.6 ^ab^	4.0 ± 0.1 ^e^	−1.5 ± 0.0 ^a^	6.5 ± 0.0 ^ab^
	90	48.3 ± 0.7 ^d^	1.8 ± 0.0 ^f^	31.2 ± 0.4 ^d^	4.3 ± 0.1 ^b^	−1.9 ± 0.1 ^g^	6.6 ± 0.0 ^ab^
**PAU6**	0	31.3 ± 0.9 ^b^	1.2 ± 0.1 ^c^	29.3 ± 0.7 ^b^	2.8 ± 0.1 ^a^	−1.0 ± 0.1 ^b^	6.6 ± 0.0 ^ab^
	30	51.2 ± 0.4 ^e^	1.3 ± 0.0 ^ac^	33.0 ± 0.2 ^c^	2.9 ± 0.1 ^a^	−1.1 ± 0.1 ^b^	6.6 ± 0.0 ^ab^
	60	52.3 ± 0.4 ^e^	1.5 ± 0.0 ^abd^	33.1 ± 0.3 ^c^	3.0 ± 0.0 ^ab^	−1.2 ± 0.0 ^bd^	6.5 ± 0.0 ^a^
	90	55.2 ± 0.7 ^f^	1.6 ± 0.0 ^bde^	32.9 ± 0.5 ^c^	3.2 ± 0.1 ^b^	−1.5 ± 0.1 ^a^	6.6 ± 0.0 ^ab^

Different letters represent significant differences (*p*-value < 0.05).

**Table 3:** Effects of *A. unedo* fruits extracts on the microbiological quality (Log_10_ cfu/g, mean ± SD (*n* = 3)) of limpets pâté samples (CTR, PAU3 and PAU6: limpets pâté with BHT, with 3% and 6% of *A. unedo* fruits extract, respectively). The results are presented as mean ± SD (*n* = 3).

Limpets Pâté	Storage Period (days)	Total Viable Mesophilic Counts	*Escherichia coli*	*Listeria monocytogenes*	*Salmonella* spp.
**CTR**	0	0.0 ± 0.0 ^a^	<10^2 a^	<10^2 a^	ND
	30	0.0 ± 0.0 ^a^	<10^2 a^	<10^2 a^	ND
	60	4.8 ± 0.1 ^d^	<10^2 a^	<10^2 a^	ND
	90	5.3 ± 0.1 ^e^	<10^2 a^	<10^2 a^	ND
**PAU3**	0	0.0 ± 0.0 ^a^	<10^2 a^	<10^2 a^	ND
	30	0.0 ± 0.0 ^a^	<10^2 a^	<10^2 a^	ND
	60	4.3 ± 0.0 ^b^	<10^2 a^	<10^2 a^	ND
	90	4.4 ± 0.1 ^b^	<10^2 a^	<10^2 a^	ND
**PAU6**	0	0.0 ± 0.0 ^a^	<10^2 a^	<10^2 a^	ND
	30	0.0 ± 0.0 ^a^	<10^2 a^	<10^2 a^	ND
	60	4.2 ± 0.0 ^c^	<10^2 a^	<10^2 a^	ND
	90	4.3 ± 0.0 ^b^	<10^2 a^	<10^2 a^	ND

ND—not detected. In each column and between lines, different letters represent significant differences (*p*-value < 0.05).

## Supplementary Materials

The following are available online at https://www.mdpi.com/2304-8158/9/6/807/s1, Table S1: Formulation of limpets (Patella spp.) pâtés samples (CTR - limpets pâté with BHT; PAU3 - limpets pâté enriched with 3% of A. unedo fruits extract and PAU6- limpets pâté enriched with 6% of A. unedo fruits extract), Table S2: Proximate composition, physicochemical properties, and antioxidant capacity (mean ± standard deviation) of Arbutus unedo fruits.
