# Supplementary material for: Impact of Aqueous Extract of Arbutus unedo Fruits on Limpets (Patella spp.) Pâté during Storage: Proximate Composition, Physicochemical Quality, Oxidative Stability, and Microbial Development
Source: Foods. 2020 Jun 19;9(6):807. doi: 10.3390/foods9060807 (PMC7353584; doi:10.3390/foods9060807)
Supplement: Supplementary file 1 [file foods-09-00807-s001.pdf]

Table S1 – Formulation of limpets (*Patella* spp.) pâtés samples (CTR - limpets pâté with BHT; PAU3 - limpets pâté enriched with 3% of *A. unedo* fruits extract and PAU6- limpets pâté enriched with 6% of *A. unedo* fruits extract).

	**CTR (g/100 g)**	**PAU3 (g/100 g)**	**PAU6 (g/100 g)**
Limpets (*Patella* sp.)	62	62	62
Water	12	12 [A]	12 [B]
Milk	10	10	10
Oil	8 [C]	8	8
Potato starch	7	7	7
Margarine	1	1	1
Salt (sodium chloride)	0.003	0.003	0.003
White pepper	0.002	0.002	0.002
Nutmeg	0.002	0.002	0.002

A – with 3% of *Arbutus unedo*; **B** – with 6% of *Arbutus unedo*; **C**– with 0.01% of BHT

Table S2 - Proximate composition, physicochemical properties, and antioxidant capacity (mean ± standard deviation) of *Arbutus unedo* fruits.

**Proximate composition**	
Moisture (g/100 g)	67.0 ± 0.2
Protein (g/100 g)	0.9 ± 0.1
Fat (g/100 g)	0.5 ± 0.0
Fiber (g/100 g)	15.4 ± 0.8
Ash (g/100 g)	0.7 ± 0.1
**Physico-chemical properties**	
Solid soluble content (%)	20.5 ± 0.2
pH	3.4 ± 0.0
L* colour parameter	53.5 ± 3.0
a* colour parameter	10.7 ± 0.8
b* colour parameter	52.1 ± 1.7
**Antioxidant capacity**	
Total phenolic content (mg GAE/100 g)	567.3 ± 27.0
DPPH radical scavenging activity (EC_{50})	0.6 ± 0.1
